# A Biogeographic Barrier Test Reveals a Strong Genetic Structure for a Canopy-Emergent Amazon Tree Species

**DOI:** 10.1038/s41598-019-55147-1

**Published:** 2019-12-09

**Authors:** Alison G. Nazareno, Christopher W. Dick, Lúcia G. Lohmann

**Affiliations:** 1 Departamento de Botânica, Universidade de São Paulo, São Paulo, SP Brazil; 2 Department of Ecology and Evolutionary Biology, University of Michigan, Ann Arbor, MI USA

**Keywords:** Evolutionary genetics, Population genetics

## Abstract

Wallace’s (1854) Riverine Barrier hypothesis is one of the earliest explanations for Amazon biotic diversification. Despite the importance of this hypothesis for explaining speciation in some animal groups, it has not been studied extensively for plant species. In this study we use a prominent Amazon tree, *Buchenavia oxycarpa* (Mart.) Eichler (Combretaceae), to evaluate Wallace’s hypothesis along the Rio Negro, a major Amazon tributary that has driven allopatric speciation for several animal taxa. We sampled six individuals from sixteen localities along both river banks, and used a modified ddRADseq protocol to identify SNP markers. Our population genomic data revealed strong genetic structure for *B. oxycarpa* sampled across banks of the Rio Negro (ϕ_CT_ = 0.576, *P* < 0.001), supporting the hypothesis that the Rio Negro acted as a significant genetic barrier for *B. oxycarpa*. Our study shows that gene flow for this large and well-dispersed Amazon tree is impeded by riverine barriers, though this has not yet resulted in speciation. Future studies focused on species with different life histories, including species restricted to non-flooded forests, are needed to further advance our understanding of Amazon rivers as drivers of biotic diversification.

## Introduction

The patterns of population structure can provide insights into the evolutionary history of individual taxa. Migration, a key evolutionary force, is expected to weaken the degree of genetic structure within a species range^1–3^, and is largely dependent on species-specific ecological traits^3–6^. For instance, ecological differences among species explains much of the interspecific variance in levels of genetic differentiation in birds across physical barriers^6^. These findings were also observed for other animal species^7–10^.

For sessile organisms such as plants, landscape features and life-history traits (e.g., associated with pollination and dispersal) can restrict migration and increase the amount of genetic variation among populations^3,4,11,12^. Indeed, strong genetic differentiation has been observed across physical barriers for plant species with restricted dispersal abilities^13–15^. For example, highland and mountain ranges acted as effective barriers to gene flow for the tree species *Styrax sumatrana*^14^, a species rarely dispersed by animals^16^. On the other hand, for plant species with high dispersal abilities, geographic barriers may not promote significant population structure^17,18^. In the Amazon Basin, where rivers are a potential cause of allopatric population differentiation for a plethora of taxa, high rates of cross-river gene flow have been documented for several different plant species^17–19^. Along the Rio Negro, one of the largest tributaries of the Amazon river broadly acknowledged as a biogeographic barrier since Wallace^20^, the first study to test this hypothesis for plants reported high rates of gene flow across river banks for two animal dispersed canopy-emergent trees^17^. A more recent population genomics study recovered weak genetic differentiation of a fish-dispersed shrub species spanning the Rio Negro^18^. Thus, extensive gene flow across large Amazon rivers may be expected for plant species with high dispersal abilities^17–19,21^. One limitation of most prior studies is that they involved species that are most common in the flooded forests and therefore able to endure flooding and benefit from seed dispersal by water. There are as yet very few studies of species that occur primarily in *terra firme* (unflooded) forest^17^.

The study species *Buchenavia oxycarpa* (Mart.) Eichler is a prominent timber tree species (up to 30 m in height) distributed in lowland *terra firme* forests of South America (Ecuador, Peru, Brazil, Venezuela and Bolivia). In Brazil, *B. oxycarpa* is found in the Amazon, Cerrado, and the Atlantic Forest^22^. In the Amazon Basin, *B. oxycarpa* occurs at relatively high densities in humid forests, often near rivers and periodically flooded terrains. Due to its high wood quality, *B. oxycarpa* has been overharvested in some areas, as in the Cerrado^23^. *Buchenavia oxycarpa* is pollinated by small bees^24^. In the Peruvian Amazon, *B. oxycarpa* is dispersed by two sympatric primate species, *Saguinus mystax* and *S. fuscicollis*^25^. Although these primate species are not found in the Central Amazon basin, it is very likely that seeds of *B. oxycarpa* are dispersed by other species of the genus *Saimiri*. The spongy mesocarp of the indehiscent fruits of *B. oxycarpa* may be associated with hydrochory, so water dispersal may be possible for this species^26^.

Even though no genetic information is available for *B. oxycarpa*, we would expect high rates of gene flow between populations of this animal-dispersed plant species based on genetic studies of tree species with similar ecology^17–19,21^. As a consequence, populations of *B. oxycarpa* located on opposite margins of the Rio Negro – a putative riverine barrier – should present weak population genetic structure. This picture may be expected since results from recent studies have indicated that long-dispersal syndromes, such as vertebrate-dispersal, are responsible for low levels of genetic differentiation for riverine plant species with widespread distributions^17^.

In this study, we aim to test the influence of the Rio Negro as a biogeographic barrier to gene flow for *B. oxycarpa* (i.e., a test of the Wallace’s Riverine Barrier hypothesis^20^). While most Amazon tree populations occur in low densities or have scattered distributions, *B. oxycarpa* is among the few plant species that are sufficiently common along both banks of the Rio Negro, allowing an adequate sampling to test whether the Rio Negro is an effective genetic barrier. To this end, we sampled *B. oxycarpa* at sixteen localities on opposite river-banks of the Rio Negro and used a modified high-throughput DNA sequencing methodology (i.e., double-digest Restriction-site associated DNA sequencing – ddRADseq)^27^ to identify anonymous SNP markers from the nuclear genome. Through this study, we hope to contribute to our current understanding about the importance of Amazon rivers for the assembly and evolution of the plant biota.

## Results

The number of single-end raw reads of 101 bp produced for each lane of HiSeq. 2000 Illumina containing 48 *B. oxycarpa* individuals ranged from 102 million to 154 million. Each read started with a barcode sequence that identified each sample (up to 10 bp long), which was followed by a 6 bp restriction site, and 85 bp of usable data. The mean number of retained reads that passed the default quality filters, including a Phred quality score > 33, and which contained an identifiable barcode, were 2,165,480 ± 124,807 SE. Throughout the *B. oxycarpa* genomes, further filtering (10-fold coverage; presence in at least 85% of the individuals; MAF > 0.01) resulted in 3,298 unlinked polymorphic SNP markers with a mean coverage depth per locus of 18.2 ± 5.6x. The minor allele frequency (MAF) averaged 0.095 ± 0.0363 SD. No significant departures from HWE were observed in any population or locus after a Bonferroni adjustment (*P* > 0.000015). In addition, no LD was observed after a sequential Bonferroni correction for k tests (k = 5.4 × 10^6^, *P* < 9.2 × 10^−9^).

We detected 278 potential loci that were under diversifying selection with the false discovery rate (FDR) set to 0.05. Thus, a total of 3,020 neutral loci were used in genomic analyses.

### Population genomic structure and the genetic barrier hypothesis

**Figure 1:**
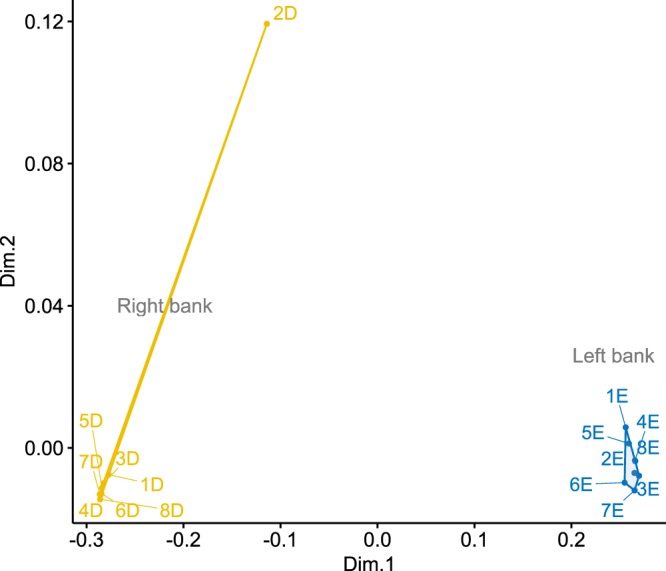
Patterns of geographical structure in *Buchenavia oxycarpa* (Mart.) Eichler represented by multidimensional scaling (MDS) of the matrix of genetic distances based on 3,020 neutral loci.

**Figure 2:**
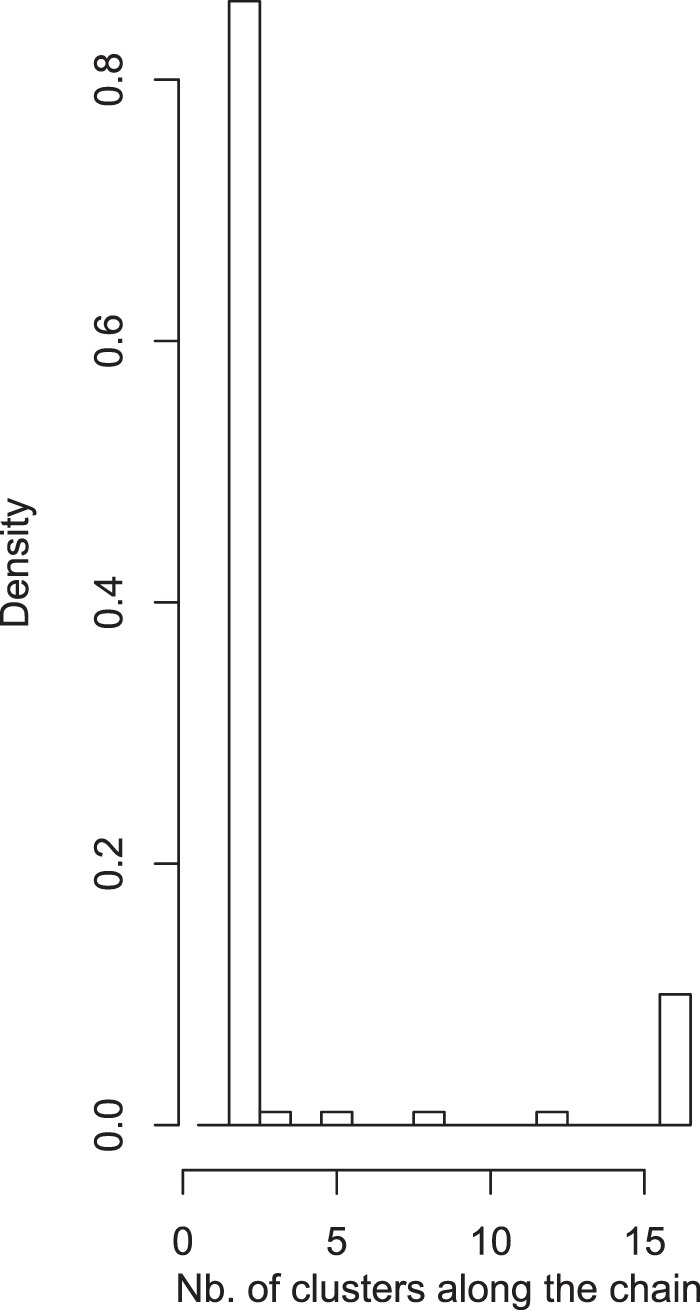
Population clustering analyses for *Buchenavia oxycarpa* (Mart.) Eichler as calculated by GENELAND showing the density of the estimate of k along the Markov chain (after a burn-in of 1000 × 100 iterations), using 3,020 neutral loci.

Using the MDS and Bayesian clustering methods, we identified one potential barrier to gene flow in the Rio Negro for both sets of SNP loci analyzed (i.e., neutral loci and loci putatively under divergent selection). An examination of the stress values (Kruskal’s stress = 0.003 for neutral loci and 0.010 for loci putatively under divergent selection) determined that two dimensions were sufficient to explain genetic patterns along the Rio Negro. The MDS plot indicated a clear separation of samples between the left and right banks of the Rio Negro (Fig. 1A,B). The genetic structure pattern from the MDS analysis closely matched that obtained using Bayesian clustering analysis. Geneland results clearly delineated two groups with minimal variance in the posterior probabilities of population estimation over multiple runs using a spatial model (Fig. 2A,B).

The 120 pairwise estimates of *F*_ST_ based on neutral loci ranged from 0.0086 to 0.6711 and all but 17 pairwise estimates were statistically significant (*P* < 0.05), indicating limited differentiation between *B. oxycarpa* population pairs from the Rio Negro (see Table S2). Based on the results of the multiple matrix regression using neutral loci exclusively, a significant portion of the variation in pairwise genetic distances is explained mainly by the historic isolation caused by the river acting as a barrier to gene flow (93.2%). Part of the genetic divergence remains unexplained (6.7%), and only a small proportion (0.1%) of that variation can be explained by the combined effect of a river barrier plus isolation by distance. Results of simple matrix correlation between genetic and geographic distance were not significant when applied separately to both banks of the Rio Negro (r = 0.274, *P = *0.191 for the right bank; r = 0.067, *P* = 0.740 for the left bank) nor when applied between pairs of *B. oxycarpa* populations on opposite banks of the river (r = 0.055, *P* = 0.663).

The hierarchical multi-locus evaluation of genetic differentiation, performed using an AMOVA, agrees with our previous results indicating that the Rio Negro can be a barrier to gene flow. Most of the genetic variation was attributable to differences observed between banks (ϕ_CT_ = 0.576, *P* < 0.001; Table 1) rather than among populations within river banks (ϕ_SC_ = 0.027, *P* < 0.001, Table 1), strengthening our findings that Rio Negro is a genetic barrier for *B. oxycarpa*.

**Table 1:** Analysis of Molecular Variance (AMOVA) based on 3,020 neutral loci for *Buchenavia oxycarpa* (Mart.) Eichler along the Rio Negro, Amazon Basin, Brazil.

	Sum of squares	Variance components	% of Variation	P-value
Between banks	14018.14	181.86	57.67	0.000
Among populations within banks	2858.94	8.48	2.69	0.000
Within populations	16881.43	125.01	39.64	0.000
Total	33758.51	315.35		

### Population tree and historical migration events

**Figure 3:**
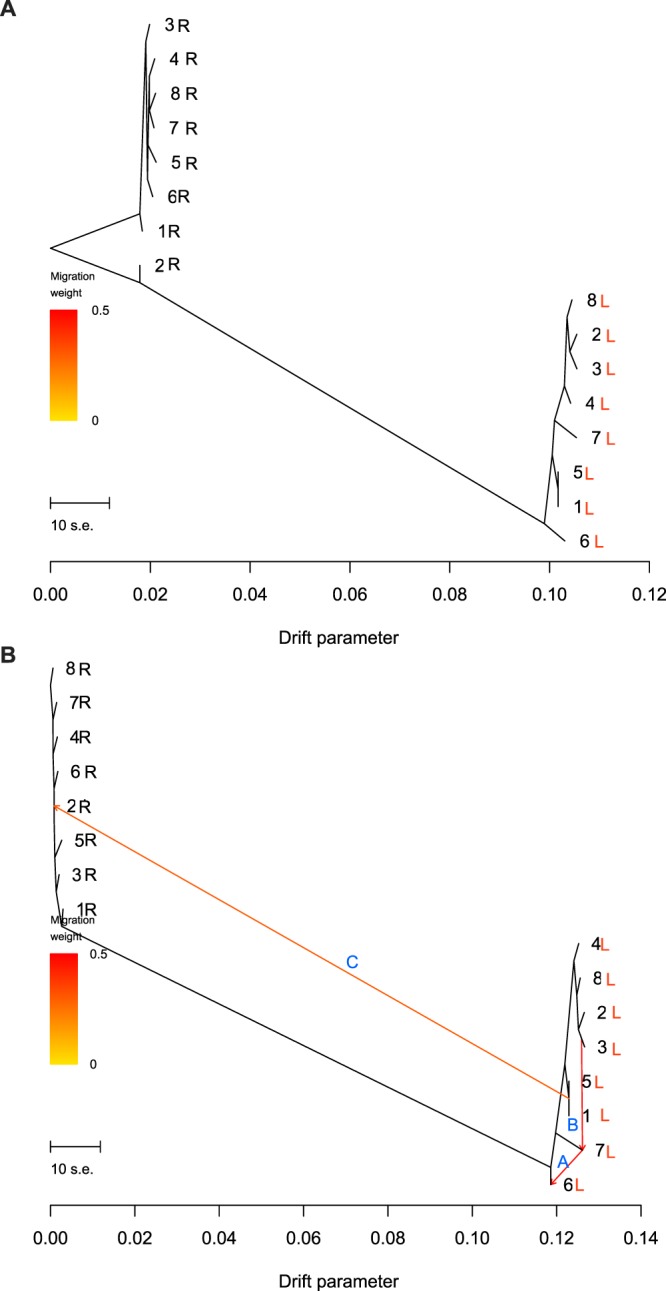
(**A**) Structure of the graph inferred by TreeMix for *Buchenavia oxycarpa* (Mart.) Eichler populations along the left (L) and right (R) banks of the Rio Negro, Amazon Basin, Brazil. (**B**) Population tree with branch lengths scaled to the amount of genetic drift between banks of the Rio Negro. Arrows indicate the inferred migration proportion and the letters, in blue, indicate the order of the migrations. The scale bar shows ten times the average standard error of the entries in the sample covariance matrix. The drift parameter reflects the amount of genetic drift that has occurred among *B. oxycarpa* populations.

The best maximum likelihood tree showed that populations on each side of the Rio Negro form two clades that correspond to the left and right river bank samples (Fig. 3A), explaining 99.49% (Fig. 3A) of the variance in relatedness among *B. oxycarpa* populations in the Rio Negro. Historical migration events (up to three events) were added sequentially to the tree. The graph model (Fig. 3B) explained 99.61% of the variance in relatedness among *B. oxycarpa* populations. The first added migration edge goes from Pop7L to Pop6L with a weight of 0.43 (Fig. 3B). Although there are migrations between *B. oxycarpa* populations within and among banks of the Rio Negro (Fig. 3B), migration events with high weights were observed for population pairs on the same bank of the Rio Negro (e.g., Pop3L-Pop7L and Pop7L-Pop6L; Fig. 3B).

## Discussion

Wallace’s Riverine Barrier hypothesis^20^ is an allopatric speciation model that aims to explain contemporary biodiversity patterns in the Amazon basin. From a reinterpetation of the Wallace’s Riverine Barrier hypothesis in population genetics terms, we can expect that large rivers, such as the Amazon, will reduce or prevent gene flow between populations on opposite river banks, ultimately leading to allopatry and species distributions that are restricted to particular interfluvial regions. A large body of studies have shown that the rivers of the Amazon Basin are important barriers to gene flow for birds^28,29^, amphibians^30^, primates^31^, and plants^18^. Although biogeographic hypotheses such as Wallace’s have often been evoked to explain diversity patterns, plants do not show the same patterns of endemism as animals^32^– leaving a major gap in our understanding of the evolutionary history of Amazon forests^33^. Furthermore, basic knowledge of the Amazon landscape remains elusive^34^. Therefore, an improved understanding of the genetic structure of Amazonian plants can help advance our understanding of the region’s geophysical history.

In this study, we tested whether the Rio Negro, a major tributary of the Amazon river and an allopatric barrier for primates, birds and amphibians^28,30,31^, is a barrier to gene flow for the tree species *Buchenavia oxycarpa*. While most Amazon tree populations occur in low densities or have scattered distributions^35^, *B. oxycarpa* is among the few plant species that are sufficiently common to allow adequate sampling along both banks of the Rio Negro. We used RadSeq to identify neutral SNP loci, which allowed for robust genetic analyses and a characterization of population genetic structure despite the relatively small population sample sizes^27^.

Contrary to what may be expected for an animal-dispersed plant species^4^, our population genetic data indicated a strong and significant population genetic structure for *B. oxycarpa* across the Rio Negro. The Bayesian and genetic distance–based clustering analyses grouped the populations located at opposite banks of the Rio Negro into two different groups (Figs. 1 and 2), showing a spatial pattern that corresponds to geographic location. All populations located on the left bank of the Rio Negro were grouped within the same cluster, while all populations located on the right bank were grouped within another cluster. These results indicate that historical gene flow via seeds and/or pollen occurred in very low frequency across banks of the Rio Negro. The AMOVA analyses reiterated these results, as the high proportion of total neutral variance attributed to the variance among banks (57.67%) indicated limited gene flow between populations on opposite river-banks separated by up to 11.7 km. These results strengthen the role of the Rio Negro as a historical barrier for *B. oxycarpa*.

The multiple matrix regression analysis using neutral SNPs indicates that 93.2% of the variation in pairwise genetic distances is explained by historical isolation caused by the riverine barrier. This analysis also indicates that geographic distance is not a primary driver of population structure for *B. oxycarpa*. In addition, no similarities between neutral genetic and geographic distance were observed within each bank of the Rio Negro, indicating that divergence with significant gene flow is feasible within each river-bank. In fact, pollen and seeds of *B. oxycarpa* seem to have been dispersed longer distances within than across river banks, with historical gene flow occurring up to 84 km at the left bank of the Rio Negro as indicated by our population graph analysis. This genetic pattern (i.e., no isolation by distance and extensive gene flow within each river bank) was also observed for *Amphirrhox longifolia* in a recent study conducted in the same location^18^.

Riverine plant species with low dispersal abilities are more likely to show hierarchical genetic structure^36,37^ than plants that can move extensively across rivers^17,18^. The patterns of neutral genetic structure observed here seem to have resulted from species-specific traits. However, this trend is not consistent with what has been observed in other animal-dispersed plant species in wider Amazon rivers. As a matter of fact, the Rio Negro does not seem to pose a barrier for a low-density and widely distributed canopy-emergent tree species (*Caryocar villosum*) that grows in *tierra firme* forests, nor to a habitat-specific tree (*C. microcarpum*) that grows in seasonally flooded black-water forests^17^. These results are expected given the long distances of gene flow associated with bat-pollination and seed dispersal by fish^17^.

In this context, the behavior and movement of frugivorous animals seems to constitute an important evolutionary force shaping patterns of genetic structure of riverine plant species. Although the specific disperser of *B. oxycarpa* is unknown in the study area, their seeds are dispersed by two sympatric primate species (*Saguinus mystax* and *S. fuscicollis*) in the Peruvian Amazon^25^. These findings suggest that other primate species (e.g., *Saimiri* spp.) may act as dispersal vectors of the seeds of *B. oxycarpa* along the Rio Negro. Although some primates can eventually cross smaller Amazon rivers (e.g., *Alouatta macconnelli*, *Sapajus apella*^31^), the Rio Negro seems to be a dispersal barrier for primates^20,31^. Indeed, Boubli and co-authors^31^ showed that this wider river represents a genetic barrier that limits the distribution of the primate genera *Ateles* and *Chiropotes* to the left bank and *Saguinus* and *Sapajus* to the right bank in the upper Rio Negro. Although water can disperse seeds to greater distances than other dispersal mechanisms^38^, the strong genetic differentiation observed for *B. oxycarpa* in opposite banks of the Rio Negro are congruent with the distribution patterns of primates. Further studies of the seed ecology of *B. oxycarpa* may confirm this observation. Studies of this nature would also shed light on the role of plant-animal dispersal mutualisms in shaping patterns of population genetic structure. Given that fruits are a key component of primate diet in the Amazon Basin^39^ and that primates are important seed dispersal vectors in tropical forests^40,41^, an improved understanding of the actual role of rivers as barriers in this region will further improve our understanding of the role of allopatric speciation for the origin and maintenance of Amazon plant diversity.

Landscape features may also play an important role in structuring genetic diversity of *B. oxycarpa*. The Rio Negro is subject to long periods of flooding^42^, especially in the lower portions where *B. oxycarpa* populations were sampled. In addition, inundation extent can vary among river banks, which can lead to differences in flowering and fruiting phenology, possibly restricting gene flow by pollen across river banks. Future studies evaluating the reproductive biology of *B. oxycarpa* in both banks of the Rio Negro may enable us to better predict genetic patterns for this species. Furthermore, river characteristics such the degree of river discharge and width can impact gene flow^18,43,44^. For instance, a recent population genomic study carried out in the core Amazon Basin showed that the strength of the riverine barrier for the plant species *Amphirrhox longifolia* depended on the width of the rivers separating populations^18^.

Although the environmental conditions, morphological, and reproductive traits of *B. oxycarpa* individuals were not recorded in our study, these types of data can help infer explicit targets of selection and adaptation, especially when correlated with allelic frequencies. Studies of this nature would be extremely valuable for species of conservation concern such as *B. oxycarpa*, which has been drastically overharvested in some regions^23^.

Overall, the population genetic structure data reported here supported the hypothesis that the Rio Negro acted as a significant genetic barrier for *B. oxycarpa*, though not enough to generate novel taxonomic diversity. Although this study provided new evidence about the role of rivers in shaping genetic structure on plants, our knowledge about landscape genetics and allopatric speciation history of Amazon plant diversity is still limited. Clearly, larger-scale population genomic studies on plants with varied ecological traits are greatly needed in order to test whether the patterns observed here are representative of the floodplain flora of the Rio Negro as a whole. Additional studies, conducted in Amazonian waterways with distinct features would complement our current understanding, which is focused on patterns observed along the Rio Negro. It would also be important to study plants that are restricted to the non-flooded *terra firme*, without any adaptations to flood tolerance. While this study focused on the analysis of historical genetic structure, detailed analyses based on contemporary gene flow would provide additional insights. More comprehensive studies are also needed to identify putative associations between ecological-environmental traits and allelic frequency. Studies of this nature would allow us to better understand how deterministic and neutral processes have contributed for the evolutionary trajectory of Amazon plant species.

## Materials and Methods

### Study area and sampling

**Figure 4:**
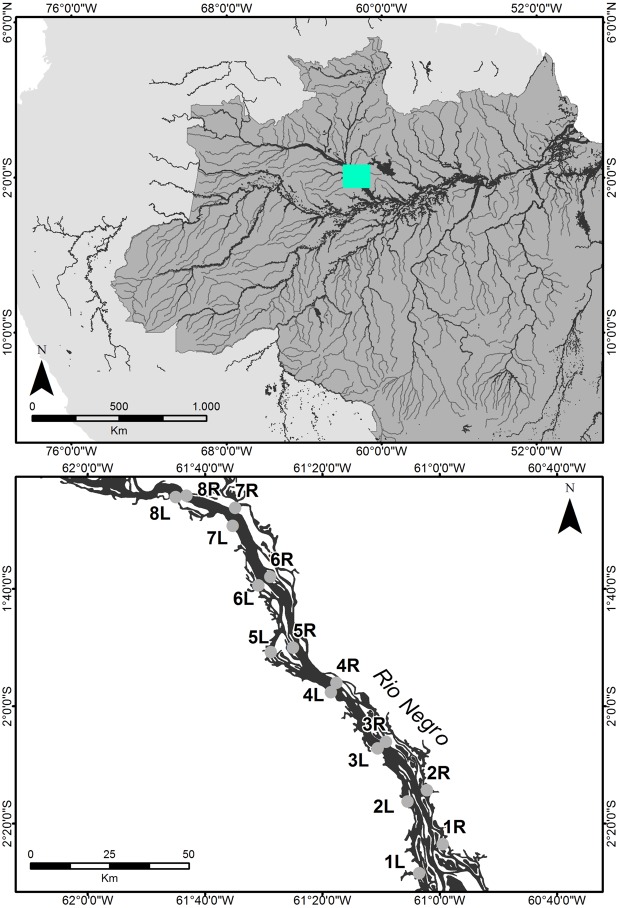
Sampling locations (1 R–8 R, 1 L–8 L) of *Buchenavia oxycarpa* (Mart.) Eichler along the left (west) and right (east) banks of the Rio Negro, Amazon Basin, Brazil.

The Rio Negro (Fig. 4) is the fifth-largest river in the world^45^. This river runs for 1700 km throughout the Amazon Basin, with the mouth of the river near the city of Manaus, Amazonas State, Brazil. Although the geological history of this river is poorly understood, geomorphological studies suggest that the mouth of the river migrated 150 km eastward to its current location^46^. The floodplain forests bordering the river, known as *igapó*, are home to several hundred tree species^47,48^ and are mainly lowland rainforests. Sub-montane, montane, and other lowland vegetation, such as white sand-soil forests^49^, also occur throughout the basin. The region has a mean annual rainfall of 2200 mm (IBGE) and the climate is classified as tropical equatorial, with a dry season occurring between June and August, and the rest of the year considered the rainy season. Because its nutrient-poor soils are unsuitable for agriculture, deforestation along the Rio Negro has been minimal.

Leaf samples of flowering *B. oxycarpa* individuals were collected during the rainy season of 2016. Sixteen sampling sites (populations) located on both the left (L) and right (R) banks of the Rio Negro were included in the study (Table S1, Fig. 4). All samples were taken from floodplains, with populations occurring within a 141 km range. Each population was paired with a corresponding population on the opposite river bank, located at distances ranging from 3.3 km (Pop3R – Pop3L, Fig. 4) to 11.7 km (Pop1R – Pop1L, Fig. 4). In each population, six individuals were sampled at intervals of at least 50 m to avoid collection from related trees. One voucher specimen was collected per population (Table S1), and all vouchers were deposited in the University of São Paulo Herbarium (SPF), São Paulo, Brazil. Because this study uses SNPs that offer high-resolution assessment of genetic structure^27,50–53^, sample sizes smaller than those normally used in population genetics studies are sufficient as the limitations inherent to small samples are offset by the large numbers of SNPs^18,54^. To validate the use of a small sample size for *B. oxycarpa*, following Nazareno *et al*.^18^, we assessed pairwise genetic differentiation by randomly reducing the number of samples from six to two. The results confirm that the sample sizes used are adequate for genetic structure analyses [*F*_ST_ (n = 6) = 0.343, 95% CI (0.292, 0.395); *F*_ST_ (n = 2) = 0.321, 95% CI (0.267, 0.376)].

### Library preparation and sequencing

The preparation of the libraries and DNA sequencing followed the protocol described in Nazareno *et al*.^18^. Briefly, we used the Macherey-Nagel kit (Macherey-Nagel GmbH & Co. KG) to extract genomic DNA from leaf samples. Two genomic libraries were constructed using a double digest RADseq protocol^55^ and following the adjustments outlined in Nazareno *et al*.^18^ related to minimizing variation in number of reads per individual. Before digestion, we used the Qubit dsDNA Assay Kit (Invitrogen) to quantify the concentrations of double-stranded DNA. Samples were adjusted to equal molar concentration, and the final DNA concentration for each sample was 500 ng.µL^1^. Two restriction enzymes, *EcoRI* and *MseI* (New England Biolabs), were used to digest each sample, and digestions were carried out in a total volume of 20 µL, using 17 µL of resuspended DNA, 5 units of EcoRI, 5 units of MseI, and 1X CutSmart buffer (New England Biolabs). The protocol consisted of 3 hours at 37 °C, with a final 20 min deactivation step at 65 °C. The Agencourt AMPure XP system (Beckman Coulter) was used to purify the reactions, following the manufacturer’s instructions, with elution in 40 µL TE buffer. The cleaned digests were quantified using Qubit to standardize the initial DNA mass to be added into an adapter ligation. Adapter ligations were conducted using a total volume of 30 µL, with 42 ng DNA, 0.22 µM of a non-sample specific *MseI* adaptor (common for all samples), 0.33 µM of a sample specific *EcoRI* double-strand adaptor for each DNA sample, 1U of T4 DNA ligase (New England BioLabs), and 1.3 X T4 ligase buffer which were incubated at 23 °C for 30 min. Restriction enzymes were then heat-killed at 65 °C for 10 min followed by a slow cooling to room temperature (23 °C).

After cleaning the reactions, ligation products were amplified in 20 µL PCRs, containing 13.5 µL of the ligation product, 0.2 µM of Illumina PCR primers, 0.2 mM dNTPs, 1.0 mM MgCl_2_, 0.5 U of iProof^TM^ High-Fidelity DNA polymerase (BIO-RAD), and 2X of iProof buffer. An Eppendorf PCR System was used for PCR using the following protocol: 98 °C for 30 s, 20 cycles of 98 °C for 20 s, 60 °C for 30 s, and 72 °C for 40 s, followed by a final extension at 72 °C for 10 min. Before pooling samples in each library, samples were purified using the Agencourt AMPure XP system and the DNA quantified using Qubit. The concentration of DNA for each sample ranged from 2.13 ng.µL^−1^ to 13.00 ng.µL^−1^. We prepared multiplexed libraries with generally the same amount of DNA per sample. The target size range to select genomic fragments was 375–475 bp, and automated size-selection was performed using a 2% agarose cartridge (Pippin Prep; Sage Science, Beverly, MA). We used the Agilent 2100 Bioanalyzer (Agilent Technologies) with the Agilent DNA 1000 Kit to measure size, quantity and quality of each individual library. Libraries were sequenced (100-bp single-end reads) at The Centre for Applied Genomics in Toronto, Canada, in a single lane (each pooled with 48 *B. oxycarpa* individuals) of an Illumina HiSeq. 2000 flow cell (Illumina Inc., San Diego, CA).

### Identifying and genotyping SNPs

The procedure used to identify and genotype SNPs is fully outlined in Nazareno *et al*.^18^. The program Stacks 1.35^56,57^ with the *de novo* assembly was used to analyze files containing the raw sequence reads. The program ustacks was used to produce consensus sequences of RAD tags, as it aligns short-read sequences from a single sample into exactly matching stacks. To estimate the diploid genotype for each individual at each nucleotide position, we used a maximum-likelihood framework^58^. The optimal minimum depth of coverage to create a stack was set to three sequences, with the maximum distance permitted between stacks set to two nucleotides, and the maximum number of stacks allowed per *de novo* locus set to three. We used an alpha value for the SNP model of 0.05. A catalog of consensus loci containing all loci and merging all alleles together was built using Cstacks; individual genotypes were then compared to the catalog using sstacks, and rxstacks was used to exclude problematic loci. Finally, we used the POPULATIONS program^56,57^ to identify the loci present in at least 85% of individuals (-r 0.85), with a minimum stack depth of 10 (-m 10), a Minor Allele Frequency (MAF) of 1% (–min_maf 0.01), and ddRAD tags requested to be present in all populations (-p 16). The final analysis included only the first SNP per locus.

### Quality control of the genomic data

For all populations, we determined the number of raw sequence reads and unlinked SNPs. We also assessed deviation from Hardy–Weinberg equilibrium (HWE) using the exact test, which is based on Monte Carlo permutations of alleles and is the most appropriate for small sample sizes^59^. HWE tests were done using the adegenet package^60,61^ implemented in R^60,61^. We used the Genepop 4.0^62^ program to test for linkage disequilibrium (LD) between loci in each population, calculating exact probabilities with a Markov Chain consisting of 100 batches and 5000 iterations per batch. After adjusting the *p* value, SNPs that failed the HWE test and SNP pairs in LD in at least seven locations (corrected for multiple k tests using the sequential Bonferroni procedure^63^) were excluded from further analyses. Based on the final dataset, we calculated minor allele frequencies for *B. oxycarpa* using the adegenet^60,61^ package in R^64^.

### Detection of outlier loci

We used the Bayescan software to identify SNP loci as having higher (divergent selection) or lower (balancing selection) levels of population divergence than strictly neutral loci^65^. This software incorporates locus and population-specific regression terms, avoiding unrealistic assumptions such as island migration models, symmetrical gene flow, or equal population sizes^65,66^. Bayescan was run with 20 pilot runs of 10,000 iterations, a burn-in of 50,000, and a final run of 100,000 iterations. We set odds of the neutral model to 10,000 (i.e., the neutral model is 10,000 times more likely than the model with selection^65^) to minimize false-positives.

### Population genomic analyses

To investigate the effects of the river on *B. oxycarpa* population structure, we assessed the genetic structure and the historical connectivity patterns between populations along and across the river using complementary genetic analyses. The methods are described in Nazareno *et al*.^19^, and a brief overview is provided below.

First, we calculated genetic distance among populations (D_A_^67^) and visualized the results by applying multidimensional scaling (MDS) in XL-STAT (Addinsoft). This was done with the Scaling by MAjorizing a COnvex Function (SMACOF) method. As an ordination technique, MDS plots populations with similar genetic structure closer together in ordination space as established by a stress factor. No assumptions relating to the cause of structure, HWE, or gametic equilibrium are required. We used the GENELAND 4.0.2^68^ package in R to develop a Bayesian model to better understand the geographic distribution of genetic variability. This approach incorporates spatial data while identifying spatially explicit genetic discontinuities, minimizing the Hardy-Weinberg and linkage disequilibrium that would result if individuals from different, randomly mating populations were incorrectly grouped. The spatial model with correlated allele frequencies proposed by Guillot *et al*.^68,69^ was used as it enables the inference of differentiation due to limited gene flow caused by physical barriers. We conducted 100 independent runs of 1,000,000 in length, discarding the burn-in of 500,000 iterations in post-processing. As the most likely number of k populations was unknown, it was treated as a simulated variable along with the MCMC simulations (1 ≤ k ≤ 16). The modal number of genetic groups of the best run (based on posterior density values) was considered as the number of genetic clusters (K).

We used ANOVA to assess pairwise genetic differentiation (*F*_*ST*_) following Weir & Cockerham^70^ and SPAGeDi^71^ to calculate *F*_*ST*_. The significance of deviation of *F*_*ST*_ values were estimated using a jackknife procedure across loci. To assess visual similarity between genetic and geographic distances based on both the MDS and GENELAND methods, we used a Mantel test^72^ for isolation by distance (IBD) to verify if the overall pattern met the expectation of decreasing genetic similarity with increasing geographic distance. To test the riverine barrier hypothesis for the Rio Negro, we deconstructed the genetic structure using a multiple matrix regression, allowing us to assess the relative contribution of long-term historical divergence and the effects of IBD. The model proposed by Legendre & Legendre^73^ was used as it evaluates the relationship across three matrices: (1) pairwise genetic distances [*F*_ST_/(1 − *F*_ST_)^74^] between *B. oxycarpa* populations; (2) Euclidian distances representing the geographic distance between pairwise *B. oxycarpa* populations; and (3) a pairwise binary matrix of isolation by the river as an expression of long-term historical divergence. This binary matrix was constructed by coding each *B. oxycarpa* population pair in relation to the river, with populations on the same river bank as ‘0’ and those on different river banks as ‘1’^27^. Multiple matrix regression and a single Mantel test were performed in R^64^ using 10,000 permutation tests of significance for the correlation coefficient.

To examine the effect of the river on genetic variation between populations, we used a nested hierarchical analysis of molecular variance (AMOVA^75^), defining two hierarchical levels of population differentiation: between populations from opposite river banks; and between populations along each bank. Arlequin 3.5.2^76^ was used to calculate population differentiation estimates and their statistical significance based on 20,000 random permutations.

To quantify historical connectivity based on neutral loci, we used TreeMix 1.12^77^ to construct historical relationships between populations based on a population graph analysis that permits population divergence and migration. Because the model used in Treemix allows for population differentiation in the presence of post-divergence admixture/migration (m), it improves the likelihood fit of a bifurcating phylogeny. The branch lengths of the resulting phylogeny are proportional to the amount of genetic drift per branch, based on a composite maximum likelihood of the local optimum tree^78^. Thus, inference is based on “shared genetic drift” between sets of populations, assuming that shared drift implies a shared evolutionary history^79^. We added stepwise migration edges, inspecting the results for consistency between runs, and we used R^64^ to visualize the population graph and residuals.

## Supplementary information

Supplementary information

## Data Availability

SNP dataset is available for download from the Dryad Digital Repository (DOI: https://doi.org/10.5061/dryad.8pk0p2nht).
