# Supplementary material for: A Biogeographic Barrier Test Reveals a Strong Genetic Structure for a Canopy-Emergent Amazon Tree Species
Source: Sci Rep. 2019 Dec 9;9:18602. doi: 10.1038/s41598-019-55147-1 (PMC6901565; doi:10.1038/s41598-019-55147-1)
Supplement: Supplementary file 1 — Supplementary information [file 41598_2019_55147_MOESM1_ESM.docx]

**A BIOGEOGRAPHIC BARRIER TEST REVEALS A STRONG GENETIC STRUCTURE FOR A CANOPY-EMERGENT AMAZON TREE SPECIES**

**Supporting Information**

ALISON G. NAZARENO^1^*, CHRISTOPHER W. DICK^2^, LÚCIA G. LOHMANN^1^*

1 Departamento de Botânica, Universidade de São Paulo, São Paulo, São Paulo, Brazil.

2 Department of Ecology and Evolutionary Biology, University of Michigan, Ann Arbor, MI, USA.

*E-mail: [alison_nazareno@yahoo.com.br](mailto:alison_nazareno@yahoo.com.br) (AGN), [llohmann@usp.br](mailto:llohmann@usp.br) (LGL)

**TABLE S1** Collection information for *Buchenavia oxycarpa* (Mart.) Eichler populations sampled in Rio Negro, Amazon Basin, Brazil.

| Population Code | River bank | Latitude | Longitude | Voucher no.* |
| --- | --- | --- | --- | --- |
| 1L | Left | 02°28'26.8" S | 61°03'26.7" W | EYK237 |
| 2L | Left | 02°16'14.7" S | 61°05'27.8" W | EYK239 |
| 3L | Left | 02°07'09.5" S | 61°10'32.5" W | Lohmann882 |
| 4L | Left | 01°57'37.3" S | 61°18'30.0" W | BMG699 |
| 5L | Left | 01°50'45.5" S | 61°28'43.9" W | BMG705 |
| 6L | Left | 01°39'22.5" S | 61°30'52.8" W | BMG707 |
| 7L | Left | 01°29'13.6" S | 61°35'17.2" W | BMG709 |
| 8L | Left | 01°24'19.4" S | 61°44'59.5" W | AFG254 |
| 1R | Right | 02°23'31.3" S | 60°59'26.9" W | EYK338 |
| 2R | Right | 02°14'17.7" S | 61°02'08.8" W | BMG719 |
| 3R | Right | 02°06'01.5" S | 61°09'07.2" W | EYK337 |
| 4R | Right | 01°56'00.1" S | 61°17'34.8" W | EYK336 |
| 5R | Right | 01°50'00.7" S | 61°25'00.1" W |  |
| 6R | Right | 01°37'56.2" S | 61°28'51.5" W | EYK334 |
| 7R | Right | 01°26'12.3" S | 61°34'49.8" W | BMG718 |
| 8R | Right | 01°24'07.0" S | 61°43'08.8" W | EYK333 |

* All specimens are deposited at São Paulo University Herbarium (SPF), SP, Brazil.

**TABLE S2** Matrix of the geographic distances (km; above diagonal) and the genetic differentiation (*F*_ST_; below diagonal) based on 3,020 neutral loci between populations of *Buchenavia oxycarpa* (Mart.) Eichler from the Rio Negro, Amazon Basin, Brazil.

|  | 1R | 2R | 3R | 4R | 5R | 6R | 7R | 8R | 1L | 2L | 3 L | 4L | 5L | 6L | 7L | 8L |
| --- | --- | --- | --- | --- | --- | --- | --- | --- | --- | --- | --- | --- | --- | --- | --- | --- |
| 1R | 0 | 18 | 37 | 61 | 78 | 100 | 124 | 136 | 12 | 17 | 37 | 59 | 81 | 100 | 120 | 138 |
| 2R | 0.0713 | 0 | 20 | 44 | 62 | 83 | 107 | 120 | 26 | 7 | 20 | 43 | 66 | 84 | 103 | 122 |
| 3R | 0.0190* | 0.0558 | 0 | 24 | 42 | 63 | 88 | 100 | 43 | 20 | 3 | 23 | 46 | 64 | 83 | 102 |
| 4R | 0.0298 | 0.0549 | 0.0096* | 0 | 18 | 39 | 64 | 76 | 65 | 44 | 24 | 3 | 23 | 39 | 59 | 77 |
| 5R | 0.0064* | -0.0665 | -0.0220 | -0.0194 | 0 | 23 | 48 | 58 | 81 | 60 | 41 | 18 | 7 | 22 | 43 | 60 |
| 6R | 0.0324 | 0.0723 | 0.0103* | 0.0113* | -0.0142* | 0 | 24 | 37 | 104 | 83 | 64 | 41 | 24 | 5 | 20 | 39 |
| 7R | 0.0232 | 0.0620 | 0.0086* | 0.0010* | -0.0171* | 0.0078* | 0 | 16 | 129 | 107 | 88 | 65 | 47 | 25 | 6 | 19 |
| 8R | 0.0254 | 0.0841 | 0.0155* | 0.0115* | -0.0076* | 0.0123* | 0.0113* | 0 | 140 | 119 | 100 | 77 | 56 | 36 | 17 | 3 |
| 1L | 0.6077 | 0.2917 | 0.6073 | 0.6054 | 0.6060 | 0.6009 | 0.6041 | 0.6087 | 0 | 23 | 41 | 63 | 84 | 104 | 124 | 141 |
| 2L | 0.6380 | 0.3712 | 0.6410 | 0.6412 | 0.6515 | 0.6346 | 0.6378 | 0.6395 | 0.0643 | 0 | 19 | 42 | 64 | 83 | 103 | 121 |
| 3L | 0.6372 | 0.3748 | 0.6402 | 0.6404 | 0.6478 | 0.6340 | 0.6373 | 0.6381 | 0.0625 | 0.0256 | 0 | 23 | 45 | 64 | 84 | 102 |
| 4L | 0.6295 | 0.3613 | 0.6316 | 0.6309 | 0.6395 | 0.6252 | 0.6285 | 0.6291 | 0.0317 | 0.0414 | 0.0192* | 0 | 23 | 41 | 61 | 79 |
| 5L | 0.6222 | 0.3398 | 0.6244 | 0.6240 | 0.6299 | 0.6178 | 0.6217 | 0.6229 | -0.0436 | 0.0706 | 0.0439 | 0.0319 | 0 | 21 | 41 | 57 |
| 6L | 0.6298 | 0.3700 | 0.6333 | 0.6322 | 0.6476 | 0.6255 | 0.6307 | 0.6292 | 0.1304 | 0.1568 | 0.1490 | 0.1453 | 0.1335 | 0 | 20 | 38 |
| 7L | 0.6481 | 0.3857 | 0.6511 | 0.6512 | 0.6711 | 0.6420 | 0.6474 | 0.6467 | 0.1241 | 0.1252 | 0.1100 | 0.1167 | 0.1164 | 0.0086* | 0 | 20 |
| 8L | 0.6304 | 0.3635 | 0.6318 | 0.6323 | 0.6371 | 0.6269 | 0.6292 | 0.6316 | 0.0490 | 0.0300 | 0.0190 | 0.0375 | 0.0441 | 0.1307 | 0.1007 | 0 |

Asterisks denote values that are not significant below the 0.05 level.
